# Women Empowered to Connect With Addiction Resources and Engage in Evidence-Based Treatment (WE-CARE)—an mHealth Application for the Universal Screening of Alcohol, Substance Use, Depression, and Anxiety: Usability and Feasibility Study

**DOI:** 10.2196/62915

**Published:** 2025-02-07

**Authors:** Krystyna Isaacs, Autumn Shifflett, Kajal Patel, Lacey Karpisek, Yi Cui, Maayan Lawental, Golfo Tzilos Wernette, Brian Borsari, Katie Chang, Tony Ma

**Affiliations:** 1 Benten Technologies Manassas, VA United States; 2 College of Behavior and Community Sciences, School of Social Work University of South Florida Tampa, FL United States; 3 University of Michigan Department of Family Medicine Ann Arbor, MI United States; 4 Center for Data to Discovery and Delivery Innovation San Francisco Veterans Affairs Health Care System San Francisco, CA United States; 5 Department of Psychiatry and Behavioral Sciences UCSF Weill Institute for Neurosciences University of California, San Francisco San Francisco, CA United States

**Keywords:** service linkage, digital health, education, mental health, substance use disorder, SUD, alcohol use disorder, chatbot, childbearing women, women, alcohol, substance use, empowerment, evidence-based treatment, usability, feasibility, mobile health, mhealth, app, depression, anxiety, screening, e-screening

## Abstract

**Background:**

Women of childbearing age (aged 18-44 years) face multiple barriers to receiving screening and treatment for unhealthy alcohol and substance use, depression, and anxiety, including lack of screening in the primary care setting and lack of support in accessing care. The Women Empowered to Connect with Addiction Resources and Engage in Evidence-based Treatment (WE-CARE) mobile app was developed to test universal screening with women of childbearing age and linkage to care after an anonymous assessment.

**Objective:**

In this study, we aimed to investigate the feasibility and acceptability of providing anonymous screening instruments through mobile phones for alcohol and substance use, as well as depression and anxiety, for women of childbearing age.

**Methods:**

We used agile development principles based on previous formative research to test WE-CARE mobile health app with women of childbearing age (N=30) who resided in 1 of 6 counties in central Florida. WE-CARE included screening instruments (for alcohol, substance use, depression, and anxiety), a moderated discussion forum, educational microlearning videos, a frequently asked questions section, and resources for linkage to treatment. Individuals were recruited using flyers, academic listserves, and a commercial human subject recruiting company (Prolific). Upon completion of the screening instruments, women explored the educational and linkage to care features of the app and filled out a System Usability Scale to evaluate the mobile health app’s usability and acceptability. Postpilot semistructured interviews (n=4) were conducted to further explore the women’s reactions to the app.

**Results:**

A total of 77 women downloaded the application and 30 completed testing. Women of childbearing age gave the WE-CARE app an excellent System Usability Scale score of 86.7 (SD 12.43). Our results indicate elevated risk for substance use in 18 of the 30 (60%) participants, 9/18 (50%) also had an elevated risk for anxiety or depression, and 11/18 (61%) had an elevated risk for substance use, anxiety, or depression. Participants reported that WE-CARE was easy to navigate and use but they would have liked to see more screening questions and more educational content. Linkage to care was an issue; however, as none of the women identified as “at-risk” for substance use disorders contacted the free treatment clinic for further evaluation.

**Conclusions:**

The mobile health app was highly rated for acceptability and usability, but participants were not receptive to seeking help at a treatment center after only a few brief encounters with the app. The linkage to care design features was likely insufficient to encourage them to seek treatment. The next version of WE-CARE will include normative scores for participants to self-evaluate their screening status compared with their age- and gender-matched peers and enhanced linkages to care features. Future development will focus on enhancing engagement to improve change behaviors and assess readiness for change.

## Introduction

Alcohol and substance use can have devastating health consequences for women. The physical health risks to women who engage in heavy alcohol and substance use include increased likelihood of injury, overdose, organ damage, and STDs due to unsafe sex practices [1]. In addition, binge drinking (defined as 4 or more drinks on one occasion in women) has reached epic proportions (44%) in young women in the United States [2,3], and the rate is increasing rapidly in women over the age of 35 [2,3]. The number of women dying of opioid overdoses has also increased by 260% between 1999 and 2017 [4]. Substance use during pregnancy can also elevate the risk of miscarriage, fetal alcohol syndrome, and birth defects [5]. The US Preventative Task Force (USPTF) reported that about 50% of US pregnancies are unplanned or unintended, with women between the ages of 18-24 facing a higher risk of unplanned pregnancies [6]. As such, alcohol and substance use during pregnancy can pose a combination of risks and potential consequences for both the mother and baby, including fetal alcohol spectrum disorders [7,8].

While the physical health risks of alcohol and substance use are well-documented, it is equally important to recognize the associated risks of mental health illness. A systematic review revealed that 20%-40% of women with an alcohol use disorder also had an anxiety disorder and showed a strong association with major depressive disorder [9,10]. In addition, women who enter treatment for substance use disorder (SUD) frequently have a personal history of domestic violence, trauma, or sexual assault [11-13]. Domestic violence, trauma, or sexual assault can all have physical consequences, on an individual’s physical health which can further impact mental health.

Universal screening for alcohol and substance use in health care settings is an effective way to identify individuals in need of help and support but is not widely implemented. Universal screening is defined as providing the same set of questions regarding alcohol and substance use to all individuals, regardless of past medical history or how the patient presents in a health care setting [14]. By implementing universal screening for all of their patients, including women of childbearing age, health care providers (HCPs) minimize the stigma associated with the inquiries. In 2016, the implementation of universal screening in primary care practices was less than 3% and less than 1% of the HCP subsequently engaged the patient in counseling [15]. Since then, USPTF has recommended universal screening for unhealthy alcohol use for all adults aged 18 years or older within primary care settings, followed by a brief conversation discussing the results [5]. This has resulted in more awareness and use of alcohol and drug screens in private practice settings, but as of 2020, private practice settings have still not met the goals set by the USPTF in 2018 [5,16]. USPTF specified the goals of their recommendation were to reduce overall rates of unhealthy alcohol use and improve individual health and social outcomes resulting from risky use through early intervention [17].

Challenges exist for implementing universal screening and supporting women of childbearing age who are at risk or with alcohol or SUD. For women of childbearing age, one such challenge included a lack of awareness of their risk of misuse as noted in the 2017 Surgeon General’s *Facing Addiction Report* [18]. Women aware of personal risk may be reluctant to speak with their HCPs out of fear induced by stigma [19,20]. In some states, women are imprisoned for substance use during pregnancy or their children can be taken away [21]. Even if the women had concerns about personal risk and were looking to make an appointment, many were faced with long wait times to receive care [22]. The National Council for Mental Health Wellbeing revealed that 43% of adults were not able to engage in substance use treatment with 28% of those individuals not being able to get an appointment shortly after requesting care [23]. Rural patients may perceive additional barriers related to living in small, close-knit communities where the confidentiality of screener results is perceived as not being as secure as in an urban setting [24-27]. Anonymous self-screening solutions can overcome many of these barriers.

Challenges also exist for HCPs to implement universal screening and support those at the various stages of risk for alcohol or SUD. One common obstacle is a lack of awareness of recommended screening tools and training on tools and substance use. While 84% of HCPs reported asking young adults about their alcohol use, and 80% of obstetrician-gynecologists asked about alcohol or substance use as part of a visit for women of childbearing age, the majority of HCPs did not use a validated screening instrument [28] such as the Tobacco, Alcohol, Prescription medication, and Other Substance Use Tool (TAPS-1 [29] and the Substance Use Risk Profile–Pregnancy (SURP-P [30]), which are valid and effective in accurately identifying those at risk for substance use [31]. As a result, it is unclear if the risk for alcohol or substance use is being identified in these encounters. In addition, the majority of HCPs are not adequately trained to administer, interpret results, and take appropriate action based on the results of the screeners [25,32]. In addition, providers in rural communities with these skills often have a heavier patient load than urban doctors and have less time for discussing the screener results, risks, and appropriate next steps [25].

Screening, Brief Intervention, and Referral to Treatment (SBIRT) can be an effective approach in the screening process and referral to treatment for individuals with alcohol or SUD [33]. SBIRT involves screening for alcohol and substance use for severity, followed by a brief intervention (eg, 10-15 minutes) and, if indicated, referral to treatment [33]. SBIRT’s effectiveness in reducing alcohol use is well supported across a variety of settings [34]. For example, when SBIRT was implemented in a trauma outpatient clinic, 59% of the patients were identified as being at risk for substance use [33]. SBIRT promotes shared decision-making [35], hinging on clinicians building trust [36], using patient-centered language to reduce stigma [37], and exploring treatment options together. Within primary care settings, SBIRT can help providers identify those suffering from substance use or mental health challenges [38], despite the benefits challenges still exist in implementing SBIRT or other screenings, referrals, and linkage to care in all care settings [39] (eg, gynecologist offices) for women of childbearing age. However, the efficacy of SBIRT’s “brief intervention” portion for drug use in primary care and emergency departments is still under consideration [40] with 1 systematic review revealing only moderate supporting evidence that interventions in the emergency department reduced alcohol-related injuries [41], another showing a reduction in visits to emergency departments following the SBIRT intervention [42], and another study showed mixed evidence surrounding the effectiveness of non–face-to-face computerized screening in the emergency department [43].

The brief intervention offered in SBIRT often incorporates motivational interviewing (MI) [44]. MI is a collaborative communication style that evokes and seeks to resolve personal ambivalence and strengthen personal commitment and motivation to change the behavior of interest [45]. The 2021 SAMHSA (Substance Abuse and Mental Health Services Administration) Advisory, *Using Motivational Interviewing in Substance Use Disorder Treatment,* emphasizes the need to discuss each individual’s readiness to change and personal history [45]. Engaging clients through the use of MI-consistent skills (eg, reflections and affirmations) is a way to evoke personal goals and values consistent with behavior change, thus increasing intrinsic motivation to achieve a healthier lifestyle. However, learning high-quality MI skills can be labor-intensive [46,47], and as such MI is difficult to provide at scale in primary care settings.

Recently, MI has been used in multiple settings and with multiple populations, including inpatient, emergency rooms, rural settings [48], and other ethnicities to reduce alcohol or substance use [49-51] and MI through text messages has been well received by adults [52,53]. MI has also been applied through digital format using text messaging to address a variety of health behaviors including tuberculosis [54], smoking [55], and alcohol use [56]. Most recently, chatbots, which are conversational tools that frequently use artificial intelligence to provide a more engaging approach, have been used in apps (SoberGrid and CHESS) to support motivational interviewing to encourage action toward recovering from substance use [57-59], but as yet no apps exist that provide screening for SUD and alcohol and related risk factors to encourage change in risky behaviors. The use of electronic screeners (eg, assessments completed on a tablet) combined with motivational interviewing, can yield more accurate self-reports [60-63].

Digital health technology may be able to address many of these challenges. Smartphone ownership rates are high among women and individuals from diverse backgrounds, with 90% of women and 91%-97% of individuals who identify as White, Black, Asian or Hispanic backgrounds having access to a smartphone [64]. Mobile apps are therefore well suited to be used to screen for risk factors in pregnancy [65] and substance use [66] and they can play a crucial role in addressing stigma for those afraid of in-person treatment [67]. With issues related to stigma and fear addressed by the anonymous status, mobile apps can encourage more women to undergo screenings, leading to early identification of at-risk individuals, improved education, and increased treatment options for HCPs [68]. Novel digital strategies to improve mental health screenings, patient engagement, and referral to treatment show preliminary effectiveness [69]. A recent report found that using tablets in the waiting room resulted in nearly twice as many individuals screening for depression than when interviewed by nursing staff [70]. Treloar and colleagues highlight that universal screenings can help reduce stigma by reducing discrimination against certain people and groups [71]. As such, mobile health apps have the potential to support universal screening by alleviating stigma, increasing awareness for change through on-demand education, and reducing fear of seeking support and care.

This study was designed to determine whether women of childbearing age will complete anonymous screenings for substance use, alcohol, depression, and anxiety from their mobile phones. We built on previous formative research and online recruitment approaches, explored the benefits of educational and evidence-based materials and the use of linkage to care options, requested feedback on the usability and acceptability of the app, and used qualitative interviews to determine what future modifications need to be implemented before commercial release.

## Methods

### Digital Health Tool Design

The Women Empowered to Connect with Addiction Resources and Engage in Evidence-based Treatment (WE-CARE) app was designed to comprise a cross-platform (iOS or Android) mobile app where the participants were asked to complete multiple mental health screeners and then invited to explore each of the accompanying app features. All participants who were assessed as being “at-risk” for either alcohol use disorder or SUD were advised they could contact a treatment center anonymously or to call the center directly. An initial prototype was tested with 30 participants drawn from the central Florida region, recruited through listserves, a company that provides research participants for a small fee, flyers, and community outreach events. Details of the final design of each of the features to be tested in the pilot are described below.

**Figure 1:**
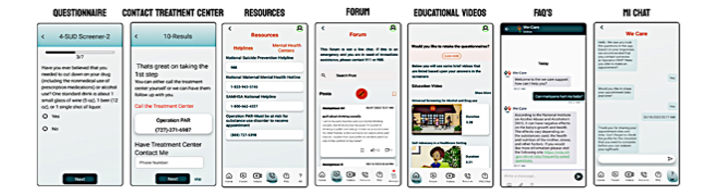
Screenshots of WE-CARE features tested in the pilot study. WE-CARE: Women Empowered to Connect with Addiction Resources and Engage in Evidence-based Treatment.

After extensive formative research [72], the following features were included for pilot testing in the mobile app: e-screenings for substance use, depression, and anxiety; anonymous self-referral to a local treatment center for further evaluation; a moderated forum for women to ask questions; a list of frequently asked questions (FAQs) to get evidence-based answers; educational videos on substance use and treatment-related information; national and local hotlines for those suffering from anxiety, depression, or a combination of the two; a chatbot to determine whether the women at risk had made an appointment with the partnered treatment center to receive a substance use evaluation; and follow up to the chatbot responses with motivational interviewing through a licensed social worker (Figure 1).

### Features Developed

#### Screening Instruments

Multiple screening instruments to be incorporated in WE-CARE were reviewed, and subject matter experts were consulted to determine the most appropriate ones for testing. In total, 4 screeners were selected to be used within the app. Immediately after downloading the app, all participants had to take all the screeners: (1) SURP-P (if the woman self-identified as pregnant); (2) TAPS-1; (3) Generalized Anxiety Disorder-2 Scale (GAD-2) for anxiety; and (4) Patient Health Questionnaire-2 (PHQ-2) for depression (Textbox 1). A binary risk assessment (elevated risk or low risk) was immediately provided to the participants who could then store the results within the app or in the phone memory (refer to Multimedia Appendix 1). The women were reminded that all their responses would be kept anonymous.

**Tools used to screen for alcohol and substance use risk, as well as anxiety and depression.**

**Substance Use Risk Profile-Pregnancy [73]**

- A 3-question tool designed for use in prenatal clinics with pregnant women to screen for alcohol, marijuana, illegal substances, and nonprescribed use of prescription medications.

**Tobacco, Alcohol, Prescription Medication, and Other Substance Use Tool [74]**

- Consists of a combined screening tool that assesses users for commonly used substances, eliminating the need for multiple screening and lengthy assessment tools.

**Generalized Anxiety Disorder-2 Scale [75]**

- A brief questionnaire to determine if someone needs additional screening for generalized anxiety.

**Patient Health Questionnaire-2 [76]**

- A simple 2-question screen to determine if someone should be considered for additional screening for depression.

If a participant was assessed as having elevated risk for alcohol or substance use, she was offered 4 choices: she could leave their phone number, but no personal information, so the treatment center could call back within 24-48 hours to provide a standardized intake, she could call a dedicated phone line and reach someone at the treatment center directly, she could call a national or local crisis hotline, or she could do nothing. If the woman was not at an elevated risk for substance or alcohol misuse but scored high for risk for depression or anxiety, she was presented with a series of crisis hotline numbers to call, as well as contact information for mental health treatment centers in one of the 6 counties of her choice.

#### Educational Microlearning Video Production

Short (1-5 min) animated cartoon videos were produced in Vyond [77] to provide information identified in the focus groups as pertinent to the women (Textbox 2). Each video topic was researched using websites at National Institute on Alcohol Abuse and Alcoholism (NIAAA), National Institute on Drug Abuse (NIDA), Substance Abuse and Mental Health Services Administration (SAMHSA), and Centers for Disease Control and Prevention (CDC). A script involving 2 or more characters was then written to provide a more naturalistic setting and conversational tone and reviewed by subject matter experts for accuracy. A Vyond editor then worked with the producer to provide costumes and a setting tailored to Florida state demographics. To add the personalization desired by the women, the videos were presented in the mobile app based on how an individual answered the screeners, (eg, if women indicated they were at elevated risk for substance use, videos about the treatment process and general information on substance use were pushed to the top of the playlist). Several new videos were created for this specific project and additional videos were repurposed from other projects related to breastfeeding for women with SUD (Textbox 2).

**Eleven short, animated videos created for educational purposes.**

**Video topics**

- What is universal screening for alcohol and drug use?
- Drug treatment center referral process.
- Information for women about marijuana use.
- Self-advocacy in a health care setting.
- Family planning.
- Breastfeeding-how to pump.
- Breastfeeding-opioid use disorder.
- Breastfeeding-marijuana use.
- Breastfeeding-tobacco cigarettes.
- Breastfeeding-prescription medications.
- Breastfeeding-alcohol use.

#### Moderated Forum

To ensure the app was providing evidence-based answers, a licensed social worker (LSW) was assigned to the moderated forum. Any participant with access to the app could make a post anonymously, but the forum was “moderated” in the sense that the LSW was scheduled to review posts each morning and would only publish ones that were relevant to the group. The LSW also could post responses to questions in the forum.

#### Frequently Asked Questions

The most popular FAQ topics and subtopics for women wanting information on substance and alcohol use were identified by querying ChatGPT 3 [78]. In the formative research stage [72], the women and HCPs expressed concern over the credibility of the information in both the moderated forum and the FAQs. As such, answers to the questions were manually compiled using source materials published at NIH, CDC, and SAMHSA, which were then reviewed by subject matter experts before being published in the FAQ section.

#### Follow-up by a Chatbot and Licensed Social Worker

A chatbot was built to engage with any individual evaluated as at-risk within 3-4 days of completing the screeners. The chatbot would ask if the person had made an appointment yet and if so, when? After discussion with the chatbot, if an appointment had not been made, the chatbot would encourage the participant to reach out to the treatment center and then a licensed social worker would follow up with the participant. The social worker would engage the participant in a manner consistent with motivational interviewing, to encourage her to make an appointment with the treatment center for further evaluation.

#### Additional Resources: National and Local Hotlines and Mental Health Facilities

Links to several national crisis lines were provided in the app such that the participant could click on the button and be immediately connected to the crisis line through her mobile phone. In addition, for those who self-identified as having either high anxiety or high levels of depression, a list of mental health centers, sorted by county, was provided within the app. All of the mental health centers were confirmed to be taking new patients before including them in the list.

### Recruitment

Upon thorough in-house testing of all of these app features, participants were recruited to test the application over a 3-month period. Participants for the pilot study were eligible to participate in the study if they were women aged 18-44 years old, able to read English at the 8th-grade level, located within central Florida (1 of 6 counties served by the treatment center), and owned a smartphone device. In addition to recruitment through listserves and contacts in central Florida, a research assistant posted flyers within the community at health care agencies, barber shops, public libraries, and recreation centers. The third approach to recruitment included inviting individuals through Prolific [79], a commercial company that recruits individuals for research projects and surveys.

Demographic information was collected through an online survey instrument embedded in the app and all participants were asked to complete a checklist of the features to test within the app before receiving compensation for participation in the research study. At the end of the app testing, all participants completed an online version of the System Usability Scale (SUS) and several open-ended questions.

### Qualitative Feedback

Upon completion of the pilot study, participants were recruited by email or through direct messaging using their Prolific IDs to participate in a virtual 1:1 semistructured interview to gather more feedback on their experience with the mobile app. Interviewers were aware of whether the woman had given the app a low or high SUS score.

### Ethical Considerations

The study was approved by the ADVARRA institutional review board (approval 00054640). All participants signed electronic informed consent documents before enrollment in the study. Participants were compensated for their time.

## Results

### Demographics

A total of 77 women downloaded the app and a total of 30 women aged 18-44 years old tested the WE-CARE mobile app. The majority of the participants were white (47%) and not of Hispanic or Latino descent (56.7%). The participant racial composition was as follows: 46.7% White, 33.3% unknown, 16.7% mixed race, and 3.3% African American. In total, 20% were of Hispanic ethnicity., 56.7% were not Hispanic or Latino, and the remaining 23% were of unknown ethnicity.

### Usability and Acceptability Score for Features Developed

**Figure 2:**
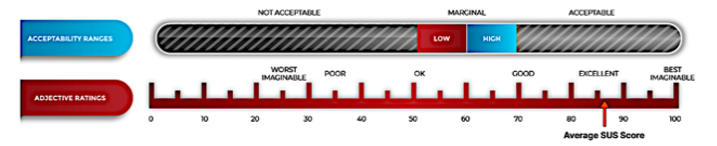
System Usability Score results for WE-CARE pilot. WE-CARE: Women Empowered to Connect with Addiction Resources and Engage in Evidence-based Treatment.

WE-CARE was well received by participants who scored it as EXCELLENT (an average SUS score of 86.7, SD 12.43). Of the individuals who scored “at-risk” for substance use, the average SUS score was slightly lower, at 84.69 (SD 12.3) while the individuals who scored “not at risk” for substance use gave the app an average SUS score of 92.73 (SD 9.14), which would have been considered “BEST IMAGINABLE” (Figure 2).

### Screening Results

While this was a usability and feasibility study, not a study designed or powered to identify risk for SUDs or mental health illness, we did examine the screening results collected in the pilot study. Out of the 30 individuals who completed the screeners, (18/30, 60%) scored at elevated risk for substance use or alcohol use behaviors, (9/18, 50%) scored at elevated risk for substance use as well as generalized anxiety, (9/18, 50%) scored elevated risk for substance use and depression, and (11/18, 61%) scored at elevated risk for substance use and either high risk for generalized anxiety or depression.

### Digital Health Tool Usage Data

#### Microlearning Video Usage

All of the women had to watch the first video (Universal Screening for Alcohol and Drug Use) but reviewing the remaining videos was optional. Using the frequency of views, it is possible to rank the videos in terms of interest, with Self-Advocacy in the Healthcare Setting being the second most popular video with 6 views. As far as the videos were concerned, participants wanted the videos to be less robotic, shorter in length, and to have more videos relating to coping with stress. Participants were asked what additional information they would like to see included in the app, and 1 participant reported they wanted to learn: “Strategies and interventions that can be used at home …. (Breathing, mindfulness exercises)”. Additional suggestions included more information on mental health issues, including “What can I do for fun or to relax instead of drinking or doing drugs? How do I tell if I have an anxiety disorder? What are some ways to reduce anxiety? How do I tell if I have a depressive disorder? What are some ways to alleviate depression?” as well as more information on self-advocacy in the doctor’s office.

#### Moderated Forum Usage

All participants were required to engage with the forum (eg, write a post or give a reaction). The forum was preseeded with some sample questions and answers, and LIKES were scattered over a variety of posts, indicating the women were reviewing the other posts. One person made an anonymous post to which the licensed social worker then responded.

#### Frequently Asked Questions Usage

The women made extensive use of the FAQs, with the majority of the main topics being explored relating to depression, anxiety, or alcohol use in both the main topics. The most popular subtopics were “How do you define a standard drink” and “How to tell if I might have an anxiety disorder? (Refer to Multimedia Appendix 2: “Number of viewings for FAQ main topics” and Multimedia Appendix 3: “Number of viewings for FAQ Subtopics” for a complete list of main topics and subtopics and their viewing frequencies).

#### Chatbot and LSW Follow-Up

Although half the individuals scored elevated risk for substance use, none of the women called or left their number with the treatment center or responded to the chatbot when it reached out to inquire whether they had made an appointment with someone at the treatment center. As such, the licensed social worker at the substance use treatment center was not able to follow up with any of the participants. During the postpilot focus group discussions, we learned one of the 4 individuals interviewed had reached out to a mental health treatment center within their area. As we were not able to track participant interactions with the mental health centers, we were not able to gauge how many of the women with at-risk scores for anxiety or depression sought additional help.

### Qualitative Feedback

A total of 4 women completed the postpilot interviews. In total, 50% of the participants were White and 50% were of more than one race. 25% of the participants were Hispanic.

In addition to providing an objective rating of the app’s usability and acceptability using the SUS scoring instrument, we asked several open-ended questions to get qualitative feedback on specific features of the application and conducted postpilot interviews. In general, the participants were complimentary about the app. One noted, “I think it's a very simplified app which I actually enjoy. It was a little nostalgic and brought me back to the earlier days of the Internet, especially with the forum included. I did think the screening tests were a little alarming because I wasn't aware I was so depressed. Overall, I enjoyed the app.”

Several participants questioned whether asking just 2 questions on either anxiety or depression was sufficient. One participant commented, “I think there needs to be additional screening questions. The issues the app wants to tackle require more than the handful of questions asked.” New features suggested by the participants included the ability to add individuals as friends through the app, making the FAQ interactive instead of a list of questions to pick from, and a person-to-person chat option.

When asked whether they had answered honestly when completing the screeners or had been “playing,” all 4 participants in the postpilot focus group said they had answered based on their own behaviors. A majority of the participants were not surprised by their scores or alarmed and felt the questions were able to accurately assess their risk for substance or alcohol use, with 1 participant reporting, “would have preferred 4-5 questions and would have been good to ask follow up – for instance, in what circumstances are you using (socially, mental coping skills)…” Another suggestion made by one of the participants involved switching the order of the questions to start with screening for depression and anxiety.

The microlearning videos received more criticism with all participants noting a mix of live-action and cartoon videos would benefit more individuals. The topics of the videos were well received with none of the individuals noting a missed topic. One participant reported, “topics were really important (esp. drinking a glass of wine”). A couple of participants felt the length of the videos was sufficient but could be shortened further to 2-3 minutes.

The FAQs did not receive much interaction from the participants, although the participants did feel having the option to look through the FAQ was beneficial to help sort through a lot of information. On the other hand, participants who reported interacting with the moderated forum felt the feature would be helpful with some additional changes such as having a live professional to contact and adding visuals to the page to make the page more “homey.”

To wrap up the discussion, questions were asked of the participants on how they viewed the motivational interviewing chatbot feature and their overall experience. The participants reported they didn’t mind the feature being a chatbot, but if a phone call needed to be made or a person was experiencing an emergency, they believed a human should step in at that point. They also desired the chatbot to provide free follow-up services and to reiterate the individual’s information will be kept confidential. Overall, participants felt they would recommend this app to another individual, one person reported, “Yes … It was marketed to the right population of people; low stakes in terms of divulging deepest darkest truths-allows a space for help that you might need.”

## Discussion

### Principal Findings

The participants scored the app very highly in terms of usability and acceptability, and enthusiasm was high for the anonymous e-screenings to be offered through a mobile phone app. SAMHSA recommends the use of the SBIRT method to combat the problem of lack of universal screening [80] and this mobile health application successfully provided the screening component of SBIRT. The data collected during the formative research period indicates that WE-CARE can support the SBIRT method by providing universal screening to women aged 18-44 years old and educational materials and referrals to partnering treatment centers, but the prototype is not yet capable of replacing the “brief intervention” component. For example, despite providing access to a free licensed counselor at the treatment center, none of the women who participated in this study were ready to seek changes in their behavior as measured by calling the treatment center for further evaluation. One participant did mention in the postpilot focus group that they did reach out to a mental health facility to address their stress or anxiety, which shows the potential of the app to help women act. The digital handshake, or linkage to care, component of the app, therefore, was not used by the participants in the pilot study. After consultation with subject matter experts, it would appear that expecting the women to seek help after completing 5 minutes’ worth of screeners on their phone was likely too high a “bar.” Instead, these results suggest that additional engagement and support would be needed to encourage the women to seek more information or treatment. Plans are underway to redesign the prototype to offer more interactions with both a chatbot and a human counselor to encourage women at risk to engage in the next steps of care.

The educational components of the app (videos, FAQs, and moderated forum) were well received. In the SUS open-ended questions and the postpilot group interviews, there was significant interest in expanding the educational materials to include additional information on coping skills and mental health (with videos, FAQs, PDFS, etc.). Other recommended additional features included creating a function to invite friends to download the app, adding a person-to-person chat option in the moderated forum, and making it possible to write in their questions for a chatbot to respond.

Given the women’s willingness to complete the screeners on their mobile phones with guaranteed anonymity, it is possible that the women would be willing to complete the screeners in a physician’s waiting room, if they could be assured of confidentiality [28,81-83]. Once a screener indicates a woman is at risk for an alcohol or SUD, the next hurdle is for the HCP to offer resources, make proper referrals, or provide suitable treatment options [84]. HCPs require training on how to effectively care for patients with alcohol or SUDs and need to understand the underlying reasons why women may engage in such behavior, such as past trauma [85]. Upon review of the screening results, the HCPs could create care plans and provide accessible mental health resources to patients based on their specific needs or refer their patients to a current list of clinics accepting new patients. If the latter option, HCPs then need to share with the patient what her journey with a care plan will look like. However, medical school training delivers minimal education in the area of substance use diagnosis and treatment [32] and HCPs have repeatedly noted they feel unprepared to offer SUD treatment or counseling [25,32]. Nonphysician or alternate health care models may be viable approaches to screening [86] and treatment and digital health technologies such as WE-CARE can help to fill in the time and knowledge gaps in the health care workforce.

In this study, over half of the women who completed the screeners in the WE-CARE app scored “at-risk” for SUD or alcohol use disorder and rates for risk for depression and anxiety in the respondents were 50% for each. The average rate for women of childbearing age meeting the *DSM-IV* (*Diagnostic and Statistical Manual of Mental Disorders* [Fourth Edition]) criteria of SUD or being in treatment in the past year for a SUD in the US is 3% for illicit drug use, 1.1% for opioid use, and 7.4% for alcohol use [87]. Similarly, in a study using the same screeners used in WE-CARE (namely the PHQ-2 for depression and the GAD-2 for anxiety), rates of depression in women of childbearing age have been reported to vary from 10% to 27% in the United States (depending on geographical location, race, and ethnicity [88,89]) and to fluctuate based on COVID prevalence [90]. The rates for anxiety in adults over 18 years of age are equally variable, peaking in 2021 at the height of the COVID-19 pandemic, at 35% [91] but before the pandemic, typically averaging around 19% [92]. Rates for anxiety and depression would be expected to be significantly higher in women of childbearing age than in the general population but gender was not reported in this study [92].

The screeners in WE-CARE were selected to identify women who are at risk for risky substance use or mental illness, not to identify substance use or mental health disorders using the criteria of a *DSM-IV* diagnosis per se. But there is a chance these rates may not be inflated. This pilot study was completed in a community that had recently experienced a major hurricane that severely impacted the citizens in 6 counties just 9 months before the study. Increased rates of alcohol and drug use [93] as well as mental illness are not uncommon after natural disasters [94]. Finally, individuals completing the WE-CARE screeners were assured of complete anonymity and therefore the screener results were not verified by individual in-person assessments. When an individual completes an assessment to report risky behavior, it has been demonstrated that the use of assessments completed on a tablet can yield more accurate self-reports [60-63]. However, self-reporting of alcohol or drug use, or concerns with mental illness, may differ in accuracy compared with when a screener is completed in front of a medical professional. The respondent may be confused about the question wording or definitions of terms, or because it was a research study, they may not have given accurate responses. Given the excellent score for usability and acceptability of the app, plans are ongoing to conduct clinical trials where the anonymous screener results will be compared with results from screeners administered by medical professionals to confirm the validity of this approach.

Mobile health apps can facilitate referrals, deliver educational materials to patients and providers, and enhance communication between patients and providers. Universal e-screenings are suitable for delivery before, during, and after an appointment and could remove the barriers associated with HCP time, stigma, willingness, or ability [95] and therefore are well-suited to the mobile health application environment. Other tailored apps exist to assist HCPs in delivering health interventions [96-103] related to substance use [96,104-106] and mental health [97,99,102] that are feasible and acceptable, but more research must be conducted to determine if the screeners are valid when delivered by a mobile health app, and also to ensure that after screening, the woman receives sufficient guidance and support to seek treatment if needed [107,108].

### Limitations

A key limitation of this study was the self-selection recruitment method used, which may have impacted the generalizability of the findings. Future studies will include samples from a larger, more diverse population. Furthermore, it is possible that only women who had concerns about their mental health opted to participate in the research study. Also, as discussed above, responses generated through self-report may be flawed if the respondent misunderstands the questions or doesn’t answer truthfully. Future versions of this tool will be tested in general clinics, where all patients will be asked to download the app as part of their annual physical and to talk with their health care provider after completing the screeners.

### Conclusions

Preliminary results on the usability and feasibility of the WE-CARE mobile health app in providing anonymous screenings for drug and alcohol use, as well as anxiety and depression, are excellent. Participants readily completed the screeners and were enthused about the opportunity to learn more about a variety of topics tailored for women in their childbearing years. Further research is necessary to validate this approach and to determine how to encourage women who score at risk for significant substance use or mental illness to seek treatment.

## Appendix

Multimedia Appendix 1 Detailed review of screeners and how they were scored in the WE-CARE app. WE-CARE: Women Empowered to Connect with Addiction Resources and Engage in Evidence-based Treatment.

Multimedia Appendix 2 Number of viewings for FAQ (frequently asked question) main topics.

Multimedia Appendix 3 Number of viewings of FAQ (frequently asked question) subtopics.

## Abbreviations

AUD: alcohol use disorder
CDC: Centers for Disease Control and Prevention
DSM-IV: Diagnostic and Statistical Manual of Mental Disorders (Fourth Edition)
FAQ: frequently asked question
GAD-2: Generalized Anxiety Disorder-2 Scale
HCP: health care provider
LSW: licensed social worker
MI: motivational interviewing
NIAAA: National Institute on Alcohol Abuse and Alcoholism
NIDA: National Institute on Drug Abuse
PHQ-2: Patient Health Questionnaire-2
SAMHSA: Substance Abuse and Mental Health Services Administration
SBIRT: Screening, Brief Intervention, and Referral to Treatment
SUD: substance use disorder
SURP-P: Substance Use Risk Profile–Pregnancy
SUS: System Usability Scale
TAPS-1: Tobacco, Alcohol, Prescription medication, and Other Substance Use Tool
USPTF: United States Preventative Task Force
WE-CARE: Women Empowered to Connect with Addiction Resources and Engage in Evidence-based Treatment
