# Supplementary material for: Women Empowered to Connect With Addiction Resources and Engage in Evidence-Based Treatment (WE-CARE)—an mHealth Application for the Universal Screening of Alcohol, Substance Use, Depression, and Anxiety: Usability and Feasibility Study
Source: JMIR Form Res. 2025 Feb 7;9:e62915. doi: 10.2196/62915 (PMC11845888; doi:10.2196/62915)
Supplement: Multimedia Appendix 1 [file formative_v9i1e62915_app1.docx]

*Multimedia Appendix 1: Detailed review of screeners and how they were scored in the WE-CARE app.*

| **Screener** | **Questions** | **Scoring System** | **At-Risk Cutoff** |
| --- | --- | --- | --- |
| SURP-P  Note, this screener will only be given to women who know they are pregnant. | 1. Have you ever smoked marijuana? 2. How many alcoholic drinks have you consumed in the month before knowing you were pregnant? 3. Have you ever believed that you needed to cut down on your drug (including the nonmedical use of prescription medications) or alcohol use? One standard drink is about 1 small glass of wine (5 oz), 1 beer (12 oz), or 1 single shot of liquor. | Count the number  of affirmative items. | Any positive result (answering YES to Questions 1 or 3, or entering a number greater than zero for Question 2) will result in the participant being referred to the alcohol and drug treatment center for further assessment. |
| TAPS - 1 | In the PAST 3 MONTHS, how often have you:   1. Used any tobacco product (for example, cigarettes, e-cigarettes, cigars, pipes, or smokeless tobacco)? 2. Had 4 or more drinks containing alcohol in one day? 3. Used any drugs including marijuana, cocaine or crack, heroin, methamphetamine (crystal meth), hallucinogens, ecstasy/MDMA? 4. Used any prescription medications just for the feeling, more than prescribed or that were not prescribed for you? Prescription medications that may be used this way include: Opiate pain relievers (for example, OxyContin, Vicodin, Percocet, Methadone), medications for anxiety or sleeping (for example, Xanax, Ativan, Klonopin) Medications for ADHD (for example, Adderall or Ritalin) | Choose one for each question:   - Daily or Almost Daily - Weekly - Monthly - Less Than Monthly - Never | If the participant answered “daily, monthly, weekly, or less than monthly” to any question besides Question 1, she will be referred to the substance use treatment center. |
| PHQ-2 | Over the PAST 2 WEEKS, how often have you been bothered by any of the following problems?   1. Little interest or pleasure in doing things? 2. Feeling down, depressed, or hopeless | 0 = not at all  1 = several days  2 = more than half the days  3 = nearly every day  2 questions; total scores range from 0 to 6. | If the participant scored 3 or higher, she will not be recommended to contact the substance use treatment center, but will be provided resources to local mental health treatment centers. |
| GAD-2 | Over the PAST 2 WEEKS, how often have you been bothered by any of the following problems?   1. Feeling nervous, anxious, or on edge 2. Not being able to stop or control worrying | 0 = not at all  1 = several days  2 = more than half the days  3 = nearly every day  2 questions; total scores range from 0 to 6. | If the participant scored 3 or higher, she will not be recommended to contact the substance use treatment center, but will be provided resources to local mental health treatment centers. |
