# Supplementary material for: Women Empowered to Connect With Addiction Resources and Engage in Evidence-Based Treatment (WE-CARE)—an mHealth Application for the Universal Screening of Alcohol, Substance Use, Depression, and Anxiety: Usability and Feasibility Study
Source: JMIR Form Res. 2025 Feb 7;9:e62915. doi: 10.2196/62915 (PMC11845888; doi:10.2196/62915)
Supplement: Multimedia Appendix 2 [file formative_v9i1e62915_app2.docx]

*Multimedia Appendix 2**: Number of viewings for FAQ main topics.*

| **FAQ Topics** | **# of viewings** |
| --- | --- |
| General Information About Depression and Anxiety | 14 |
| General Facts on Alcohol Use | 13 |
| General Information About the Impact of Alcohol and Substance Use on a Child’s Health | 6 |
| What are Risky Behaviors Related to Alcohol and Drug Use | 4 |
| General Information about Family Planning | 3 |
| General Information About Fetal Alcohol Spectrum Disorders (FASD) | 3 |
| General Information About Domestic Violence | 3 |
| General Information about Family Planning | 2 |
| General Information About Stigma | 2 |
| General Information About Treatments for Alcohol and Substance Use | 2 |
| How Does COVID-19 Effect on Drinking Habits, Substance Use, and Mental Health | 1 |
