# Supplementary material for: Women Empowered to Connect With Addiction Resources and Engage in Evidence-Based Treatment (WE-CARE)—an mHealth Application for the Universal Screening of Alcohol, Substance Use, Depression, and Anxiety: Usability and Feasibility Study
Source: JMIR Form Res. 2025 Feb 7;9:e62915. doi: 10.2196/62915 (PMC11845888; doi:10.2196/62915)
Supplement: Multimedia Appendix 3 [file formative_v9i1e62915_app3.docx]

Multimedia Appendix 3: Number of viewings of FAQ sub-topics.

| **FAQ Sub-Topics** | **# of viewings** |
| --- | --- |
| How do you define a standard drink? | 7 |
| How to tell if I might have an anxiety disorder? | 4 |
| What is considered binge drinking for women? | 3 |
| How does drug or alcohol use affect sexual behaviors? | 3 |
| How is depression usually displayed in women? | 3 |
| What role does stigma (or shame and discredit) play in women receiving treatment for alcohol? | 2 |
| What if I did not know I was pregnant and had been drinking heavily on the weekends? | 2 |
| What is considered heavy drinking for women? | 2 |
